# Tissue preserving non-invasive physical plasma treatment for cervical squamous intraepithelial neoplasia grade 3—a prospective randomized, controlled clinical trial

**DOI:** 10.3389/fmed.2025.1669933

**Published:** 2025-11-07

**Authors:** Melanie Henes, Sabine Matovina, Robert Rottscholl, You-Shan Feng, Antonia Fleischhacker, Guilin Wang, Markus Enderle, Peter Awakowicz, Sara Y. Brucker, Martin Weiss

**Affiliations:** 1 Department of Women’s Health, University of Tübingen, Tübingen, Germany; 2 Department of Pathology and Neuropathology, University of Tübingen, Tübingen, Germany; 3 Institute for Clinical Epidemiology and Applied Biometry, University of Tübingen, Tübingen, Germany; 4 Erbe Elektromedizin GmbH, Tübingen, Germany; 5 Institute for Electrical Engineering and Plasma Technology, Ruhr-Universität Bochum, Bochum, Germany; 6 NMI Natural and Medical Sciences Institute, University of Tübingen, Reutlingen, Germany

**Keywords:** cervical cancer, high-grade squamous intraepithelial lesions, cervical intraepithelial neoplasia, non-invasive physical plasma, low thermal argon plasma devitalization, tissue-preserving treatment, fertility preservation, minimally invasive therapy

## Abstract

**Introduction:**

High-grade squamous intraepithelial lesions (HSIL), such as cervical intraepithelial neoplasia grade 3 (CIN3), are precursors to invasive cancer. Although cancer develops in only 1–2 out of 10 patients with CIN3, all patients typically undergo invasive procedures. This overtreatment affects approximately 90% of CIN3 patients, especially young women, posing risks to fertility and pregnancy outcomes. Non-invasive physical plasma (NIPP) treatment via low thermal argon plasma devitalization (APD) technology offers a novel, outpatient alternative with potential tissue-preserving and antineoplastic properties.

**Methods:**

This prospective, monocentric, randomized, controlled phase IIb trial (NCT04753073) evaluated the efficacy of APD in achieving histological remission of CIN3, compared to the natural course in an untreated control group. Forty premenopausal women aged 18 years or older with confirmed CIN3 were enrolled and randomized into two groups: 20 underwent a single APD treatment session followed by large loop excision of the transformation zone (LLETZ) 6–8 weeks later, and 20 served as untreated controls undergoing LLETZ only. Pain perception and patient satisfaction were assessed via visual analog scale and the Freiburg Index of Patient Satisfaction (FIPS), respectively. Statistical analyses included Fisher’s exact tests and odds ratio (OR) calculations and were conducted using SPSS.

**Results:**

Complete histological remission of CIN3 was observed in 33.3% of APD-treated patients compared to 5.0% in the control group (*p* = 0.025, OR = 9.43). Partial remission occurred in 27.8% of APD patients and 15.0% of controls, while persistent CIN3 was more common in controls (80.0% vs. 38.9% in APD-treated patients). APD treatment also facilitated R0 resection during consecutive LLETZ in 94.4% of cases versus 65.0% in the control group (*p* = 0.082). No severe adverse events were reported, and patient satisfaction was comparable between groups.

**Conclusion:**

APD treatment demonstrates significant efficacy in inducing histological remission of CIN3, reducing lesion severity, and preserving tissue. This innovative approach offers a promising, minimally invasive alternative to conventional surgical methods, particularly for women of childbearing age. Given the current issue of overtreatment with invasive procedures, APD could significantly reduce unnecessary interventions. Larger, multicenter trials are warranted to confirm these findings and establish APD as a standard treatment for HSIL.

**Clinical trial registration:**

https://www.clinicaltrials.gov/study/NCT04753073, identifier NCT04753073.

## Introduction

Cervical cancer is the fourth most common cancer in women with claiming approximately 350,000 deaths in 2022 (1). The pathogenesis of this disease follows a progression from precancerous lesions, predominantly caused by persistent infection with high-risk human papillomavirus (hrHPV). Notably, the highest incidence rates occur in low- and middle-income countries, disproportionately affecting younger women (1). Cervical intraepithelial neoplasia (CIN), a precursor to invasive cancer, is classified into grades based on the severity of cellular dysplasia. While CIN1 exhibits mild dysplastic changes, CIN2 and CIN3 demonstrate moderate to severe alterations. Furthermore, the standardized Bethesda Classification, distinguish between low-grade squamous intraepithelial lesion (LSIL), corresponding to mild dysplasia (CIN1), and high-grade squamous intraepithelial lesion (HSIL), encompassing moderate to severe dysplasia (CIN2 and CIN3) with higher malignant potential (2). According to current guidelines, CIN3 consistently requires appropriate excisional treatment, even though only about 12% of CIN3 lesions progress to invasive cervical cancer (3). Current standard treatment for HSIL often involves large-loop excision of the transformation zone (LLETZ). In selected cases, alternative methods such as thermal, laser, or cryoablation are employed (4). LLETZ and alternative treatments typically require local or general anesthesia and result in tissue destruction, which may lead to significant complications (5, 6). These include reduced fertility, increased risk of preterm birth, higher cesarean section rates and low birth weight in subsequent pregnancies (7–9). This overtreatment with invasive procedures (relevant for approximately 9 out of 10 patients) represents a significant issue for affected women and the healthcare system. A non-invasive tissue preserving treatment, eliminating the need for general and local anesthesia, could address these challenges. Non-invasive physical plasma (NIPP) treatment via argon plasma devitalization (APD) technology using the VIO3/APC3 electrosurgical argon plasma device (Erbe Elektromedizin, Tübingen, Germany) emerges as a promising candidate. This monopolar surgical method, utilizing an low temperature argon plasma beam, enables precise, tissue-preserving and effective treatment of CIN lesions while preserving underlying stromal tissue structures by applying a homogeneous, brush-like plasma application (10–12). Antineoplastic effects are driven by reactive oxygen and nitrogen species (ROS/RNS) that exhibit transmucosal activity, ideal for eradicating precancerous lesions without damaging adjacent tissues (10, 13–17). Preliminary monocentric dose-finding and subsequent confirmatory studies demonstrated safe and effective tissue preservation in CIN1/2 patients (10, 18).

In this prospective, monocentric, controlled clinical study, we evaluate the efficacy of the APD intervention in patients with histologically confirmed CIN3, compared the outcomes to the spontaneous remission rate observed in a control cohort, and thereby aim to establish a proof-of-principle.

## Methods

### Study design

The study has been carried out as a controlled, randomized, prospective, phase IIb clinical trial (NCT04753073), performed at the Department for Department for Women’s Health, Tübingen, Germany. The trial was conducted in accordance with “The Code of Ethics of the World Medical Association” (Declaration of Helsinki) and was approved by the Ethical Committee of the Medical Faculty of the Eberhard-Karls-University Tübingen (929/2020BO1). CIN3 (HSIL) was histologically confirmed during routine examination by colposcopy-directed biopsy before study inclusion. The authors have obtained both informed consent and ethics committee approval for studies on patients, patient records, or volunteers.

### Inclusion and exclusion criteria

Inclusion criteria for study participation included premenopausal women aged over 18 years, a histologically confirmed diagnosis of CIN3, and complete visibility of the entire transformation zone and lesion margins. Exclusion criteria included incomplete visualization of the transformation zone or endocervical disease, clinical suspicion of invasive or microinvasive carcinoma, severe systemic diseases, or pregnancy. The study included only premenopausal women ≥ 18 years, as this represents the primary reproductive age group with high rates of completely visible transformation zones (T1/T2), criteria not met by postmenopausal patients.

### Patient treatment

Patients underwent clinical evaluation, including colposcopy and visual inspection with acetic acid (VIA) and Lugol’s iodine staining, to detect CIN3 lesions. Treatment was performed using low thermal APD (also known as non-invasive physical plasma (NIPP) under colposcopic guidance with the VIO3/APC3 electrosurgical system (Ref. 10160-000 and Ref. 10135-000) and 3.2 mm FiAPC (Ref. 20132-222) probes (precise APC setting, effect 1), applied at a rate of 30 s per square centimeter with a reusable silicone electrode mat. (Ref. 20183-016, all Erbe Elektromedizin GmbH, Tübingen, Germany). The APD probe was maneuvered over the targeted tissue using controlled “brush stroke” movements to minimize localized heating. The procedure was conducted on an outpatient basis without the requirement for local or general anesthesia.

### Study design

**FIGURE 1:**
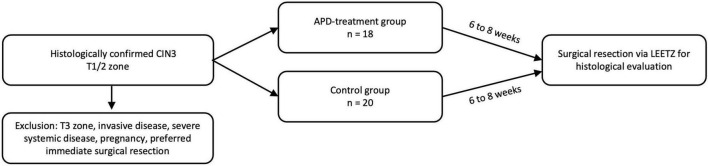
CONSORT flow diagram showing participant enrollment, randomization, follow-up, and analysis.

The primary endpoint of the statistical analysis was to compare the histological complete remission rates of CIN3 following LLETZ performed within 8 weeks after APD treatment with those of an untreated control group. The study included 20 patients in the interventional group and 20 in the control group. Two participants from the treatment group did not complete the study and were excluded from the analysis. The primary endpoint focused on assessing the rate of complete histological remission of CIN3 in the APD-treated group after subsequent LLETZ (Figure 1). The objective was to determine whether APD treatment enhances histopathological remission compared to the natural progression of cervical intraepithelial neoplasia.

### Histology, cytology, and HPV assessment

Cytological smears were routinely stained according to Papanicolaou at the Immunocytology lab at the Department for Women’s Health at the Eberhard Karls University Tübingen, reports were generated according to the Munich III nomenclature. Histological and immunohistological staining (HE and p16 monoclonal antibody at 1:2,000 titration, Abcam Ab108349) was performed according to standard protocols at the Department for Pathology and Neuropathology at the Eberhard-Karls University Tübingen on formalin-fixed, paraffin-embedded (FFPE) biopsy samples and resection specimen. The resections were processed according to the national guideline on cervical cancer. For HPV-diagnostics the Hybrid Capture 2 assay (HC2; Qiagen Inc., Hilden Germany) and p16 immunohistology (monoclonal antibody at 1:2,000 titration, Abcam Ab108349) were used at the Department for Pathology and Neuropathology and the Department of Medical Virology at the Eberhard-Karls-University Tübingen, respectively.

### Questionnaire and Freiburg index of patient satisfaction

For the acquisition of possible perception of pain following APD treatment we used a visual analog scale from 0 to 10. Scores of 0 and 1 were defined as “no pain,” 2–4 as “mild pain,” 5–7 as “moderate pain” and 8–10 as “severe pain.” Other medical conditions could be indicated as free text. Assessment of treatment satisfaction after APD intervention the “Freiburg index of patient satisfaction” was applied (19).

### Statistical analysis

The objective was to investigate whether APD induces more histopathological complete remissions compared to the spontaneous course of cervical intraepithelial neoplasia in patients treated only with the standard operative procedure (LLETZ). As this was a randomized controlled trial with proper randomization and intent-to-treat analysis, unadjusted comparisons between groups provide valid effect estimates without requiring further statistical adjustment for potential confounders. All statistical comparisons were conducted between the control and APD groups. Categorical outcomes were analyzed using contingency tables, with χ^2^ tests applied for null-hypothesis testing when cell counts were sufficient (*n* > 5); Fisher’s exact test was used when cell counts were low (*n* ≤ 5). For continuous variables, Student’s *t*-test and Mann-Whitney U test were used to test the null hypothesis between control and APD groups for normally distributed and skewed data, respectively. Normality was assessed through visual inspection of histograms and examination of skewness and excess kurtosis statistics. Within the APD group, paired comparisons of pre- and post-treatment results were conducted using paired *t*-tests or Wilcoxon signed-rank tests, depending on the distribution of the variable of interest. Unadjusted odds ratios (OR) were calculated to assess the likelihood of complete remission between control and APD (reference) groups. Due to the very small number of patients without remission (only seven total, with only one in the control group), no formal null-hypothesis testing was applied to the odds ratio, and multivariable modeling with covariate adjustment was not feasible. The randomized design of the trial ensures that the unadjusted odds ratio remains a valid and interpretable measure of treatment effect. All statistical analyses were conducted using IBM SPSS Statistics Version 28.0.0.0 (SPSS Inc., Chicago, United States), with two-sided *p* < 0.05 considered statistically significant.

## Results

From 04/2021 to 05/2024 we included 40 patients with histologically confirmed CIN3 lesions at the Dysplasia Center of the Department for Women’s Health, Tübingen, Germany in a prospective, randomized, controlled clinical trial (NCT04753073). The effect of APD treatment was compared to spontaneous clinical course in the untreated control group by histopathological examination of LLETZ samples. Following a diagnosis of CIN3, 20 patients in the treatment group underwent a single APD treatment session, followed by LLETZ excision 6–8 weeks later. In the control group, 20 patients were treated exclusively with LLETZ, performed 6–8 weeks after the diagnosis of CIN3. Two patients in the treatment group withdrew their consent to participate in the study and were excluded from the statistical analysis.

### Patient characteristics

In this study, we included women over 18 years of age with histologically confirmed CIN3. Complete visibility of the entire transformation zone (T1/2), including the margins of high-grade intraepithelial lesions, was required for participation. Exclusion criteria included incomplete visualization of the transformation zone or endocervical disease, evidence of invasive disease, severe systemic diseases, pregnancy or a preference for immediate surgical resection. Patients were informed of the experimental nature of APD application, which had demonstrated promising effects in treating CIN 1/2 lesions (18). Written informed consent was obtained in accordance with the approved ethical protocol (929/2020B01) before initiating APD or control treatment. Table 1 presents the characteristics of APD-treated patients compared to the control group. At baseline, 5.6% of patients in the APD group and 5.0% in the control group had normal cytological results (PAP I or PAP II-a). In the APD group, 27.9% were classified within the PAP III (D1/D2/-p) group compared to 50.0% in the control group, while 66.7% of the APD group were diagnosed with PAP IVa-p compared to 35.0% in the control group. 94.4% of APD treated patients and 100.0% in the control group were hrHPV-positive at baseline. In one APD case (5.6%) HPV testing lacked technical evaluability. Generally, no clinically relevant differences of baseline parameters were observed between the two groups.

**TABLE 1:** Patients characteristics APD group versus control group.

Patients characteristics		APD (*N* = 18)	Control (*N* = 20)	*p*-value
Age, years (*n* = 40, mean, range)		33.4 (22–41)	35.3 (23–47)	0.305▪
Gravidities (number, percentages per group)	0 G	9 (50.0%)	9 (45.0%)	0.039○
	I G	7 (38.9%)	2 (10.0%)	
	II G	1 (5.6%)	6 (30.0%)	
	III G	0 (0.0%)	2 (10.0%)	
	IV G	1 (5.6%)	0 (0.0%)	
	V G	0 (0.0%)	1 (5.0%)	
Parities (number, percentages per group)	0 P	11 (61.1%)	10 (50.0%)	0.013○
	I P	6 (33.3%)	1 (5.0%)	
	II P	1 (5.6%)	8 (40.0%)	
	III P	0 (0.0%)	1 (5.0%)	
Cytology (n, %)	PAP I or II-a	1 (5.6%)	1 (5.0%)	0.478○
	PAP II-p	0 (0.0%)	2 (10.0%)	
	PAP IIID1	1 (5.6%)	2 (10.0%)	
	PAP IIID2	1 (5.6%)	3 (15.0%)	
	PAP III-p or PAP III-g	3 (16.7%)	5 (25.0%)	
	PAP IV a-p	12 (66.7%)	7 (35.0%)	
HPV high risk (n,%)	unknown	1 (5.6%)	0 (0.0%)	0.876○
	positive	17 (94.4%)	20 (100.0%)	

For analysis of patient characteristics different statistical test were used and marked respectively: *T*-test (▪) was used with metric, normally distributed variables. Due to small cell counts, Fisher’s exact test (○) was used for ordinal variables.

### Primary outcome: histological assessment of APD efficacy

**FIGURE 2:**
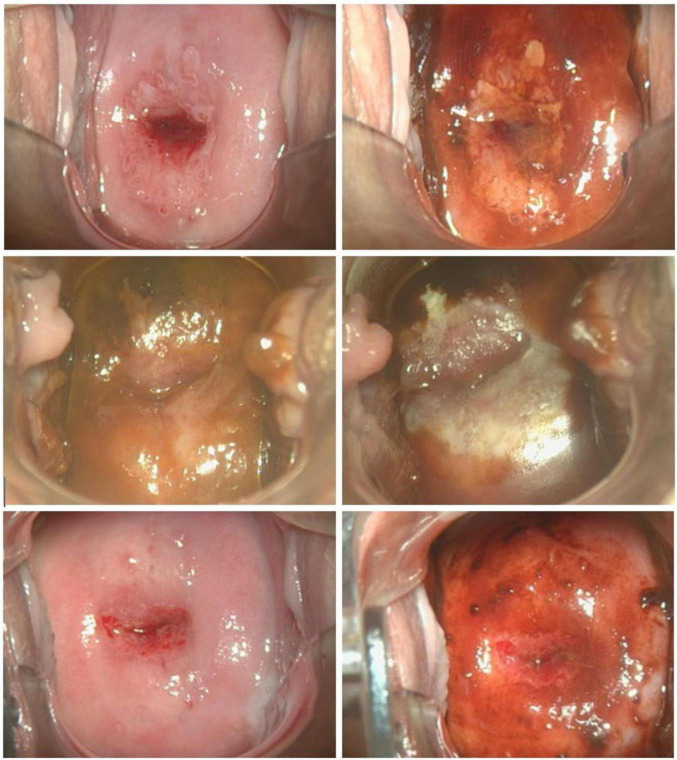
Representative colposcopic images at initial colposcopic examination (upper row) performing VIA (left) and iodine staining (right); at APD intervention (middle row) before (left) and after (right) APD treatment; at preoperative assessment 8 weeks after APD treatment (bottom row) performing VIA (left) and iodine staining (right).

The evaluation of APD efficacy was based on histological examination conducted after the follow-up LLETZ procedure, performed approximately 6–8 weeks after the initial APD treatment. Tissue samples obtained through LLETZ were analyzed to determine the presence or remission of CIN3. Histological characterization was conducted including HE in conjunction with serial sections and p16 staining in all resection specimen. Among the APD-treated patients, six cases (33.3%) demonstrated complete remission with no evidence of intraepithelial lesions, compared to only one case (5.0%) in the control group. Partial remission, including the presence of CIN1/2, was observed in 5 patients (27.8%) in the APD group, compared to 3 patients (15.0%) in the untreated control group. In contrast, histological confirmation of persistent CIN3 was found in the majority of control group patients (16 cases, 80.0%), whereas only 7 patients (33.3%) in the APD-treated group showed similar findings (*p* = 0.025). The odds ratio (OR) for achieving histopathological complete remission was calculated as 9.43 in the APD-treated group compared to the control group, indicating a markedly higher likelihood of remission with APD treatment. Table 2 summarizes the histological outcomes following APD treatment in comparison to the control group. Figure 2 illustrates a representative colposcopic image from an APD-treated patient demonstrating histological complete remission.

**TABLE 2:** Histological remission rates after APD treatment.

Histological remission		After LLETZ		
		APD (*n* = 18)	Control (*n* = 20)	
Histological characterization (n,%)	No CIN	6 (33.3%)	1 (5.0%)	*p*-value 0.025 ○
	CIN1	2 (11.1%)	0 (0.0%)	
	CIN2	3 (16.7%)	3 (15.0%)	
	CIN3	7 (38.9%)	16 (80.0%)	
CIN changes compared to study start (n,%)	Full remission	6 (33.3%)	1 (5.0%)	*p*-value 0.028 ○
	Partial remission	5 (27.8%)	3 (15.0%)	
	Persistence	7 (38.9%)	16 (80.0%)	

Statistical significance was tested due to small cell counts via Fisher’s exact test (○; two-sided).

### Secondary outcome: resection margins (R1 or R0)

The effect of APD treatment on resection outcomes after LLETZ was analyzed, focusing on its ability to reduce the severity and extent of CIN lesions. APD treatment resulted in a reduction in both the grade and size of CIN, facilitating a higher likelihood of achieving R0 resection during subsequent LLETZ procedures. Among patients in the APD-treated group, 17 out of 18 (94.4%) achieved R0 resection, characterized by the absence of dysplastic lesions at the surgical margins, compared to 13 out of 20 patients (65.0%) in the control group. This indicates a potential benefit of APD treatment in improving surgical outcomes, especially when R1 and Rx may be clinically considered as indication for post-resection (*p* = 0.045). A detailed overview of resection status distribution is provided in Table 3.

**TABLE 3:** Resection margins after LLETZ ± APD-treatment, statistical significance was determined via Fisher’s exact test (○; two-sided) due to small cell counts.

Resection margins		After LLETZ		
		APD (*n* = 18)	Control (*n* = 20)	
R-Status (n,%)	R0	17 (94.4%)	13 (65.0%)	*p*-value: 0.082 ○
	R1	1 (5.6%)	3 (15.0%)	
	Rx	0 (0.0%)	4 (20.0%)	

### Secondary outcome: evaluation of patient-reported outcomes

One notable advantage of APD treatment is its suitability for outpatient care, eliminating the need for local and general anesthesia. To assess patients’ subjective pain perception and satisfaction with the treatment, the Freiburg Index of Patient Satisfaction (FIPS) was utilized. FIPS measures the overall satisfaction with treatment, including the perceived treatment success. Higher values indicate greater satisfaction, while lower values suggest dissatisfaction. The results of the survey are summarized in Table 4. There was no statistically significant difference in satisfaction ratings between APD treatment and the standard operative procedure (LLETZ). Interestingly, we found a statistically significant improvement in patient satisfaction regarding therapeutic load of LLETZ after administered APD treatment (Supplementary Tables 1–3).

**TABLE 4:** Evaluation of patient’s satisfaction via Freiburg index (FIPS), displayed with mean ± Standard deviation (○) statistical significance was tested using *t*-test (▪), using Mann-Whitney-U-Test (*) and Wilcoxon.

Cohort	APD-treatment	LLETZ	
APD-treatment mean ± SD	1.5 (± 0.6)	1.7 (± 0.8)	*p* = 0.279 ▪/ *p* = 0.191*
Control-group	–	2.3 (± 1.1) ○	
		*p* = 0.030 ▪ /*p* = 0.033 *	

*T*-test (▪), Wilcoxon-Test for paired comparison, Mann-Whitney-U-Test for subgroup comparison (*).

No acute dose-limiting toxicities were observed during APD treatment and LLETZ. Study participants reported only mild adverse events, including smear bleeding, localized discomfort, and increased vaginal discharge, following both APD treatment and LLETZ. There were no statistically significant differences in the frequency of adverse events between the two groups. All reported side effects resolved spontaneously without the need for additional surgical intervention. An overview of the reported adverse events is presented in Table 5, with multiple responses allowed.

**TABLE 5:** Adverse events after APD-treatment and LLETZ.

Adverse events		APD-treatment	LLETZ (APD group)	LLETZ (control group)
Smear bleeding	Yes	5 (31.3%)	7 (41.2%)	8 (47.1%)
	No	11 (68.8%)	10 (58.8%)	9 (52.9%)
Increased discharge	Yes	2 (12.5%)	3 (17.6%)	0 (0.0%)
	No	14 (87.5%)	14 (82.4%)	17 (100.0%)
Local discomfort	Yes	2 (12.5%)	3 (17.6%)	1 (5.9%)
	No	14 (87.5%)	14 (82.4%)	16 (94.1%)
Any adverse event		7 (43.8%)	10 (58.8%)	9 (52.9%)

## Discussion

This prospective, randomized, controlled clinical trial demonstrates that a single APD treatment session significantly induces histological remission of CIN3 lesions compared to spontaneous remission. Complete histological remission was achieved in 33.3% of APD-treated patients versus 5.0% in the control group (*p* = 0.025, OR = 9.43). Partial remission occurred in 27.8% of APD patients compared to 15.0% of controls, while persistent CIN3 was observed in 38.9% of APD-treated patients versus 80.0% in controls. Additionally, APD treatment facilitated R0 resection in 94.4% of subsequent LLETZ procedures compared to 65.0% in controls. No severe adverse events were reported, and patient satisfaction assessed via the Freiburg Index of Patient Satisfaction remained comparable between groups (19). HSIL, corresponding to CIN2 and CIN3 according to the Bethesda Classification, represent precursors to invasive cervical cancer with higher malignant potential (2). Current standard treatment for CIN3 involves excisional procedures such as LLETZ, which achieve high cure rates but carry risks of fertility complications and adverse pregnancy outcomes (7–9). Our findings represent the first randomized controlled evidence for non-invasive plasma therapy in CIN3 treatment. Previous studies have demonstrated APD efficacy in CIN1/2 lesions, with our group reporting successful tissue preservation in lower-grade dysplasia (10, 18). The 33.3% complete remission rate observed in our study compares favorably to the 5–15% spontaneous regression rates typically reported for untreated CIN3 in historical cohorts (3). Unlike conventional ablative methods such as cryotherapy or laser ablation (4), APD operates as a monopolar surgical method utilizing argon plasma coagulation technology (11, 12) through reactive oxygen and nitrogen species (RONS) generation, inducing targeted cellular responses without thermal tissue destruction (10, 13–17).

These results suggest APD could address the significant overtreatment issue in CIN3 management, where currently all patients undergo invasive LLETZ procedures, although only 12% would progress to invasive cervical cancer over several years if left untreated, meaning approximately 8–9 out of 10 patients receive unnecessary invasive treatment (3). For women of childbearing age, APD offers a tissue-preserving alternative that avoids fertility-related complications associated with excisional treatments, including preterm delivery, premature rupture of membranes, and low birth weight (7–9). The outpatient applicability without anesthesia requirements reduces healthcare costs and patient burden compared to conventional treatments requiring local or general anesthesia (5, 6). The improved R0 resection rates following APD pretreatment suggest potential combination approaches, allowing for smaller, less invasive LLETZ procedures when surgical intervention remains necessary. Patient satisfaction, as measured by standardized instruments, remained high, supporting the acceptability of this novel approach (19). However, larger multicenter trials are required before clinical implementation, and long-term follow-up data are essential to confirm sustained remission and rule out delayed progression.

Future research should focus on optimizing treatment parameters, including energy dosing, treatment duration, and potential for repeat applications, building upon established dose-response relationships (20–24). Multicenter randomized controlled trials with larger patient cohorts are warranted to validate these proof-of-concept findings. Mechanistic studies investigating the molecular pathways underlying APD-induced remission, including effects on cell growth, cell cycle regulation, metabolism, DNA integrity, and apoptosis, could inform treatment optimization (15, 20–24). Extension to other HPV-related dysplasias, including vulvar (VIN) and vaginal (VAIN) intraepithelial neoplasia, represents logical research progression given the transmucosal efficacy of plasma treatment. Long-term follow-up studies are crucial to assess durability of remission and monitor for delayed adverse effects. Additionally, cost-effectiveness analyses comparing APD to standard treatments would inform healthcare policy decisions in line with WHO recommendations for comprehensive cervical cancer control (25, 26).

Study strengths include the randomized controlled design with histopathological confirmation of outcomes, providing objective assessment of therapeutic efficacy. The use of standardized treatment protocols based on established argon plasma coagulation principles (10–12, 18) and systematic follow-up enhances result reliability. Patient-reported outcome measures using validated instruments (19) provide comprehensive assessment of treatment acceptability. However, several limitations must be acknowledged. The single-center design and small sample size (*n* = 40) limits statistical power, generalizability and preclude multivariate analysis. The short follow-up period (6–8 weeks) cannot assess long-term remission durability or delayed recurrence. Further long-term outcomes such as recurrence rates, fertility effects, and obstetric outcomes remain unknown. Interindividual variation in cervical surface anatomy and CIN III lesion characteristics, along with operator-dependent factors, affect the reproducibility of APD treatment. The experimental nature may have introduced selection bias, and blinding was not feasible due to the treatment’s visible nature. These factors highlight the need for larger, multicenter phase III trials with extended follow-up to confirm efficacy, safety, and long-term benefits before APD can be adopted as a standard treatment for CIN3/HSIL. As an ablative method, APD does not provide histological specimens for definitive assessment, necessitating subsequent LLETZ for endpoint evaluation.

## Conclusion

APD treatment demonstrates significant efficacy in inducing histological remission of CIN3 lesions while preserving cervical tissue. This innovative approach offers a promising minimally invasive alternative for women of childbearing age, potentially reducing the current overtreatment burden in cervical dysplasia management. The findings support progression to larger multicenter trials to validate clinical implementation potential. Given the global impact of cervical cancer (1, 25, 26) and the need for tissue-preserving treatments, APD represents a valuable addition to the therapeutic armamentarium for cervical dysplasia management according to current classification systems (2).

## Data Availability

The original contributions presented in the study are included in the article/Supplementary material, further inquiries can be directed to the corresponding author.
