# Supplementary material for: Tissue preserving non-invasive physical plasma treatment for cervical squamous intraepithelial neoplasia grade 3—a prospective randomized, controlled clinical trial
Source: Front Med (Lausanne). 2025 Nov 7;12:1669933. doi: 10.3389/fmed.2025.1669933 (PMC12637225; doi:10.3389/fmed.2025.1669933)
Supplement: Supplementary file 1 [file Data_Sheet_1.docx]

**Supporting information**

**Supplementary Table 1: Survey of pain perception according to visual analog scale following APD treatment.**

| Intensity of pain or discomfort (n=16) | | | **No pain** | **Mild pain** | **Moderate pain** | **Severe pain** |  |  |
| --- | --- | --- | --- | --- | --- | --- | --- | --- |
|  | **Prior treatment** | | 15 (93.8 %) | 1 (6.3 %) | - | - |  |  |
|  | **During treatment** | | 5 (31.3 %) | 5 (31.3 %) | 4 (25.0 %) | 2 (12.5 %) |  |  |
|  | **After 4 h** |  | 12 (75.0 %) | 3 (18.8 %) | 1 (6.3 %) | - |  |  |
|  | **After 2 days** |  | 14 (87.5 %) | 2 (12.5 %) | - | - |  |  |
|  | **After 1 week** |  | 14 (87.5 %) | 2 (12.5 %) | - | - |  |  |

**Supplementary Table 2: Survey of pain perception according to visual analog scale after APD treatment followed by sequential LLETZ.**

| Intensity of pain or discomfort (n=17) | | | **No pain** | **Mild pain** | **Moderate pain** | **Severe pain** |  |  |
| --- | --- | --- | --- | --- | --- | --- | --- | --- |
|  | **Prior treatment** | | 16 (94.1 %) | 1 (5.9 %) | - | - |  |  |
|  | **During treatment** | | 10 (58.8 %) | 5 (29.4 %) | 2 (11.8 %) | - |  |  |
|  | **After 4 h** |  | 13 (76.5 %) | 1 (5.9 %) | 1 (5.9 %) | 2 (11.8 %) |  |  |
|  | **After 2 days** |  | 10 (58.8 %) | 4 (23.5 %) | 3 (17.6 %) | - |  |  |
|  | **After 1 week** |  | 12 (70.6 %) | 4 (23.5 %) | - | 1 (5.9 %) |  |  |

**Supplementary Table 3: Survey of pain perception according to visual analog scale following LLETZ alone.**

| Intensity of pain or discomfort (n=17) | | | **No pain** | **Mild pain** | **Moderate pain** | **Severe pain** |  |  |
| --- | --- | --- | --- | --- | --- | --- | --- | --- |
|  | **Prior treatment** | | 15 (88.2 %) | 1 (5.9 %) | 1 (5.9 %) | - |  |  |
|  | **During treatment** | | 15 (88.2 %) | - | 2 (11.8 %) | - |  |  |
|  | **After 4 h** |  | 9 (52.9 %) | 6 (35.3 %) | 1 (5.9 %) | 1 (5.9 %) |  |  |
|  | **After 2 days** |  | 8 (47.1 %) | 5 (29.4 %) | 3 (17.6 %) | 1 (5.9 %) |  |  |
|  | **After 1 week** |  | 11 (64.7 %) | 6 (35.3 %) | - | - |  |  |
